# Assessment of Variation in Bacterial Composition among Microhabitats in a Mangrove Environment Using DGGE Fingerprints and Barcoded Pyrosequencing

**DOI:** 10.1371/journal.pone.0029380

**Published:** 2012-01-11

**Authors:** Daniel F. R. Cleary, Kornelia Smalla, Leda C. S. Mendonça-Hagler, Newton C. M. Gomes

**Affiliations:** 1 Department of Biology and CESAM, Universidade de Aveiro, Aveiro, Portugal; 2 Julius Kühn-Institut, Federal Research Centre for Cultivated Plants, Braunschweig, Germany; 3 Institute of Microbiology, Federal University of Rio de Janeiro, Rio de Janeiro, Brazil; Institute for Genome Sciences - University of Maryland School of Medicine, United States of America

## Abstract

Here, we use DGGE fingerprinting and barcoded pyrosequencing data, at six cut-off levels (85–100%), of all bacteria, *Alphaproteobacteria* and *Betaproteobacteria* to assess composition in the rhizosphere of nursery plants and nursery-raised transplants, native plants and bulk sediment in a mangrove habitat. When comparing compositional data based on DGGE fingerprinting and barcoded pyrosequencing at different cut-off levels, all revealed highly significant differences in composition among microhabitats. Procrustes superimposition revealed that ordination results using cut-off levels from 85–100% and DGGE fingerprint data were highly congruent with the standard 97% cut-off level. The various approaches revealed a primary gradient in composition from nursery to mangrove samples. The affinity between the nursery and transplants was greatest when using *Betaproteobacteria* followed by *Alphaproteobacteria* data. There was a distinct secondary gradient in composition from transplants to bulk sediment with native plants intermediate, which was most prevalent using all bacteria at intermediate cut-off levels (92–97%). Our results show that PCR-DGGE provides a robust and cost effective exploratory approach and is effective in distinguishing among a priori defined groups.

## Introduction

Ever since Antony van Leeuwenhoek for the first time observed living microbial cells [1], studies of microbes and their interactions with the environment and other organisms have depended on the technology available to scientists. Following his discovery, the search for new methodologies and tools to improve our access to the microbial world has never ceased. In the past few decades, the development of nucleic acid based analyses of microbial communities has allowed us, for the first time, to overcome the bias of cultivation dependent methods, which has been deemed the “Great plate count phenomenon” [2], [3]. Compared to cultivation dependent methods, molecular techniques, such as 16S rRNA gene clone libraries and molecular microbial community fingerprints [denaturing and temperature gradient gel electrophoresis (DGGE and TGGE), single-strand conformation polymorphism (SSCP), and terminal-restriction fragment length polymorphism (T-RFLP)], have enabled scientists to obtain more realistic information about microbes in the environment. In a study of bacterial diversity in chronic wounds Dowd et al. [4] noted that culturing failed to identify major populations that were found using molecular methods (pyrosequencing and DGGE). In addition to this, culturing can take several days before the bacteria can be successfully identified while molecular PCR based methods can take only a few hours [4].

DGGE, since it was introduced into microbiology by Muyzer and colleagues [5], has been used to analyse the composition of a range of microbial groups, including viruses and microbial eukaryotes [6], [7]. It is still the most widely applied molecular technique for profiling the structure of bacterial communities [4], [8], [9]. Prior to the advent of next-generation sequencers, such as Roche 454 pyrosequencing, these fingerprint techniques provided the most reliable and complete overview of the community structure, diversity and dynamics of microbes [5], [10], [11]. A perceived problem with community fingerprint approaches based on universal primers, targeting higher taxonomic levels (e.g., *Bacteria*, *Archaea* and *Fungi*) is that it only reveals the abundant taxa in a set of samples [12], although this problem can be minimized by utilization of taxon specific PCR-DGGE [13]. Bands in DGGE gels, furthermore, can represent multiple species or, in contrast, the same species may be represented by multiple bands [4], [14]. The combination of taxon specific primers in a nested PCR followed by amplicons fingerprinting is often used in microbial ecology studies. This approach can profile less abundant microbial populations by narrowing the range of target microbial groups [11], [12]. However, the specificity of this approach has been questioned when low abundant taxon groups are profiled in environments containing diverse communities [11]. The reliability and resolution of group specific nested approaches still needs to be evaluated in the light of more thorough molecular microbial analyses, such as pyrosequencing approaches.

Pyrosequencing analysis provides a much more in depth and accurate estimate of microbial diversity than other molecular methods such as DGGE or T-RFLP [4], [15]. Currently, the general consent is that pyrosequencing technologies will rapidly replace conventional molecular fingerprint techniques rendering them essentially obsolete. For instance, depending on the sequence effort, barcoded pyrosequencing of 16S rRNA gene amplicons allows microbiologists to access the diversity and composition of microbial communities with a high level of resolution in virtually any environment on earth [16]. There may, however, be situations when a combined approach, i.e., using fingerprinting and pyrosequencing, may prove to be a better tactic given the much lower cost per sample and rapidity of obtaining results associated with the former technique. Pyrosequencing, furthermore, enables us to evaluate the reliability and power of resolution of classical molecular tools used for microbial community profiling.

Here, we use DGGE fingerprinting and barcoded pyrosequencing data to study compositional variation in the bacteria of distinct mangrove microhabitats, namely the rhizospheres of nursery-raised transplants and native plants and non-rhizosphere bulk sediment in a mangrove habitat in addition to the rhizosphere of nursery plants in which the transplants were raised. In a previous publication (17), we used pyrosequencing data with a focus on bacterial taxa and their perceived ecological functions in mangrove microhabitats and a nursery used to raise mangrove seedlings for reforestation projects. Here our main goal is to test whether different molecular methods (DGGE fingerprinting and barcoded pyrosequencing), taxa (*Bacteria*, *Alphaproteobacteria* and *Betaproteobacteria*) and cut-off levels (for pyrosequencing data) yields congruent results. Our specific objectives were: 1) to use DGGE fingerprinting and barcoded pyrosequencing data, at six cut-off levels (85–100%), of *Bacteria*, *Alphaproteobacteria* and *Betaproteobacteria* to assess composition in the rhizosphere of nursery plants and nursery-raised transplants, native plants and bulk sediment in a mangrove habitat; 2) to assess to what degree results obtained with the first objective are significantly congruent. Given the above, we discuss the use of PCR-DGGE as a rapid and reliable proxy for studying compositional variation in samples of highly complex microbial communities, such as those obtained from a mangrove environment [17], [18]. When validated, such an approach could minimise the costs associated with analysing several samples and provide a fast and reliable global view of microbial communities prior to pyrosequencing.

## Materials and Methods

### Sampling and total community DNA extraction

Sampling and total community DNA extraction followed Gomes et al. [17]. Briefly, four composite replicates of bulk sediment (∼20 cm of top sediment with 4 cm diameter) samples and roots of individual mangrove plants (four replicates each of *R. mangle* from nursery, transplants and natives) were sampled. During sampling, samples were treated as previously described in Gomes et al. [19] for sediment samples. Total community DNA (TC-DNA) extraction was performed from microbial cell pellets retrieved from sediment and rhizosphere samples as previously described in Gomes et al. [19].

### PCR-amplification of 16S rRNA gene fragments and DGGE analyses

Amplified 16S rRNA gene fragments suitable for DGGE fingerprint analyses of bulk and rhizosphere sediment samples were obtained after a nested approach as described previously [11]. Briefly, the amplicons obtained in the first PCR were diluted (1∶20) and used as a template for a second PCR (25 cycles) with bacterial DGGE primers F984-GC and R1378 (∼473 bp) according to Heuer et al. [12]. The sequence variation covered by these primers (*Escherichia coli* position 968–1401) is located in the hypervariable regions V6 to V9.

A nested-PCR approach (25 thermal cycles) was also applied for amplification of 16S rRNA genes of *Alphaproteobacteria* and *Betaproteobacteria* groups as previously described [13]. The amplicons obtained with group-specific PCR were diluted (1∶20) and used as a template for DGGE PCR-amplification as described above.

DGGE of the amplified 16S rRNA gene sequences was performed using the Dcode System (Universal Mutation Detection System, Biorad). The GC-clamped amplicons were applied to a double gradient polyacrylamide gel containing 6–9% acrylamide [11] with a gradient of 26–58% of denaturant. The run was performed in 1× Tris-acetate-EDTA buffer at 58°C at a constant voltage of 220 V for 6.0 h. The DGGE gels were silver-stained according to Heuer et al. [20].

### DGGE and Pyrosequencing data processing

The DGGE gels were transmissively scanned and the digitalised profiles were analysed with the Gelcompar 4.0 program (Applied Maths). The band positions and their corresponding intensities (surface) from each soil treatment were exported to a spreadsheet as previously described [18]. The band position and its intensity were used as a parameter to specify a category (DGGE band type) and relative abundance (peak area) within the sample profile, respectively [21]. The DGGE data processing resulted in a square matrix containing the presence and abundance of DGGE band types per sample.

All sequence reads analysed in this study were generated in a previous study [17] and can be downloaded from the NCBI Short Read Archive, accession number SRA023845. For the present study, we reprocessed the sequence data using the QIIME (Quantitative Insights Into Microbial Ecology) software packagepipeline (http://qiime.sourceforge.net/; checked 2011-12-09) on a computer using the BioLinux operating system (http://nebc.nerc.ac.uk/tools/bio-linux/bio-linux-6.0; checked 2011-09-30). With QIIME, we used the default arguments in the split_libraries.py function with the exception of a minimum sequence length of 150, after primer trimming, and removal of both forward and backward primers using the ‘truncate only’ argument. For the selection of OTU's, we used the ‘uclust’ method in the pick_otus.py function and the ‘rdp’ method [22] to taxonomically classify OTU's using the assign_taxonomy.py function. Chimeric sequences were identified using the ‘blast fragments’ method in the parallel_identify_chimeric_seqs.py function. We, however, modified the taxonomy depth in the last function to 3 as opposed to the default 4, so that chimera were considered chimeric if fragments were assigned to different classes or higher taxonomic levels (e.g., phyla). Our motivation for this was the observation that numerous OTU's were dubiously classified as chimeric when using a depth of 4. Blasts (http://blast.ncbi.nlm.nih.gov/Blast.cgi) of a number of these so-called chimera also revealed hits suggesting that they were not in fact chimeric.

### Statistical Analyses

DGGE and pyrosequencing matrices were constructed for (1) all *Bacteria* (2) *Alphaproteobacteria* only and (3) *Betaproteobacteria* only at the six previously mentioned cut-off levels (only for pyrosequencing). In order to visualise variation in composition with distance we used metric (Principal coordinates analysis; PCO) and nonmetric multidimensional scaling using the cmdscale() function in the R base package on Bray-Curtis distance matrices obtained with the vegdist() function in vegan on log_10_ (*x*+1) transformed OTU matrices of samples from nursery, transplanted and naturally occurring *Rhizophora mangle* plants and from bulk sediment (Bul). Both ordination techniques, however, yielded very similar results and only the PCO is shown in this paper. Analyses were performed separately for all bacteria, *Alphaproteobacteria* only and *Betaproteobacteria* only using the six previously mentioned cut-off values and DGGE band data. The Bray-Curtis distance (similarity) is frequently used in ecological work [23]–[27]. We tested for significant variation in OTU composition among microhabitats using the adonis() function in vegan [28], which performs an analysis of variance with distance matrices using permutations. In an adonis, distance matrices are partitioned among sources of variation; in this case microhabitats. In each adonis() analysis, the Bray-Curtis distance matrix of OTU composition was the response variable and microhabitat the independent variable. The number of permutations was set at 999; all other arguments used the default values set in the function.

We used Procrustes superimposition to assess to what extent pyrosequencing data using different cut-off levels and DGGE data yield similar results with respect to variance in bacterial composition among samples. Procrustes superimposition minimises the sum of squared distances between pairs of data observations in two matrices by adjusting size, rotation and translation. This squared distance is known as the Procrustes distance. Procrustes superimposition can be used to compare alternative solutions based on ordinations such as multidimensional scaling [28]. In the present study, we used the procrustes() function in vegan to visually assess congruence among PCO ordinations based on pyrosequencing data using different cut-off levels and with DGGE band data; default values were used for the arguments in the procrustes() function. This included scaling, which adjusts one configuration ‘Y’ to maximum similarity with another configuration ‘X’. The scaling is non-symmetric given that Y is scaled to fit X. In addition to the procrustes() function, the protest() function in vegan was used to estimate the significance of the Procrustes statistic. The latter function uses a statistic (r = sqrt(1-ss)) derived from the symmetric Procrustes sum of squares ‘ss’ and calls the procrustes() function a given number of times (1000 permutations in the present case).

## Results

Pyrosequence reads (16S rRNA gene sequences) were clustered into operational taxonomic units (OTUs) across a range of cut-off levels. It is often assumed that different cut-off values applied in the cluster analysis of 16S rRNA gene sequences can be used for sequence assignment to rank taxon groups. For example, while sequences with ∼97% similarity are assigned to the same species, those with ∼95% similarity are assigned to the same genus [29]–[30]. Statistical analyses of the molecular microbial community data revealed highly significant differences in the composition of *Bacteria*, *Alphaproteobacteria* and *Betaproteobacteria* among microhabitats when using DGGE profiles and pyrosequencing data at all six cut-off levels (P<0.001, Table 1 and see Figure S1, Figure S2, Figure S3 and Figure S4). In line with our previous study [17], the taxonomical classification of the OTU's (Figure S5) indicates that some bacterial groups have stronger associations with specific microhabitats. Our results show that, while *Alphaproteobacteria* and *Betaproteobacteria* were more abundant in the nursery and transplants, *Deltaproteobacteria* showed a stronger association with native plants and bulk sediment. *Epsilonproteobacteria* was more abundant in mangrove rhizospheres (transplants and natives).

**Table 1:** Results of adonis analyses with the Bray-Curtis distance matrix of OTU composition as the response variable and microhabitat the independent variable.

Species	Cut-off	F	P	R^2^
*Bacteria*	85%	F_3,14_ = 6.47	P<0.001	0.638
	91%	F_3,14_ = 4.64	P<0.001	0.559
	92%	F_3,14_ = 4.41	P<0.001	0.546
	95%	F_3,14_ = 3.41	P<0.001	0.482
	97%	F_3,14_ = 2.67	P<0.001	0.421
	100%	F_3,14_ = 1.22	P<0.001	0.250
	DGGE	F_3,14_ = 3.91	P<0.001	0.516
*Alphaproteobacteria*	85%	F_3,15_ = 5.33	P<0.001	0.57
	91%	F_3,15_ = 5.58	P<0.001	0.582
	92%	F_3,15_ = 5.39	P<0.001	0.57
	95%	F_3,15_ = 4.25	P<0.001	0.52
	97%	F_3,15_ = 3.00	P<0.001	0.43
	100%	F_3,15_ = 1.29	P<0.001	0.24
	DGGE	F_3,15_ = 8.46	P<0.001	0.679
*Betaproteobacteria*	85%	F_3,15_ = 5.64	P<0.001	0.585
	91%	F_3,15_ = 5.82	P<0.001	0.592
	92%	F_3,15_ = 5.85	P<0.001	0.594
	95%	F_3,15_ = 5.06	P<0.001	0.559
	97%	F_3,15_ = 3.96	P<0.001	0.497
	100%	F_3,15_ = 1.67	P<0.001	0.295
	DGGE	F_3,15_ = 5.67	P<0.001	0.586

**Figure 1:**
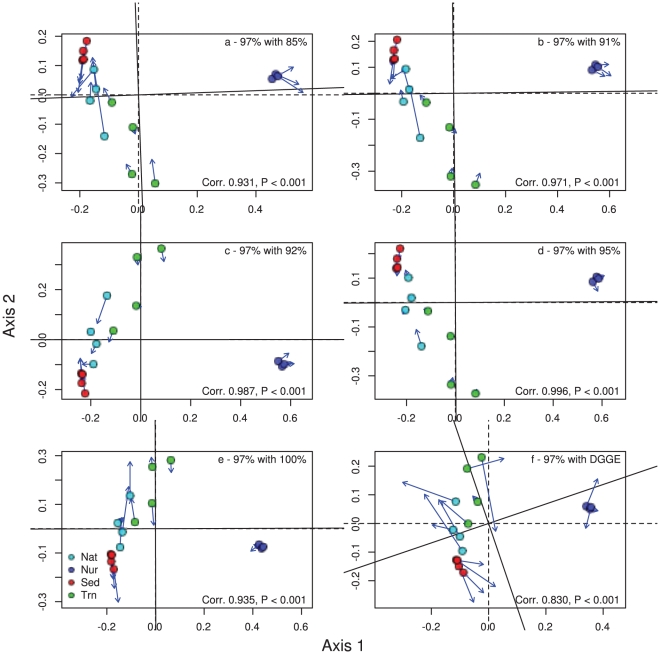
Ordination showing the first two PCO axes of the all bacteria analysis for a) 85% cut-off level with 97%, b) 91% cut-off level with 97%, c) 92% cut-off level with 97%, d) 95% cut-off level with 97%, e) 100% cut-off level and f) DGEE data with 97% cut-off level. The arrows in the ordination point to the target configuration, the actual symbols represent the rotated configuration, i.e., the ordination based on the 97% cut-off values. Correlation (Corr) and significance values are given in the lower right corner of each graph. Both target and original rotated axes are shown as crosshairs on the plot using unbroken and dashed lines.

**Figure 2:**
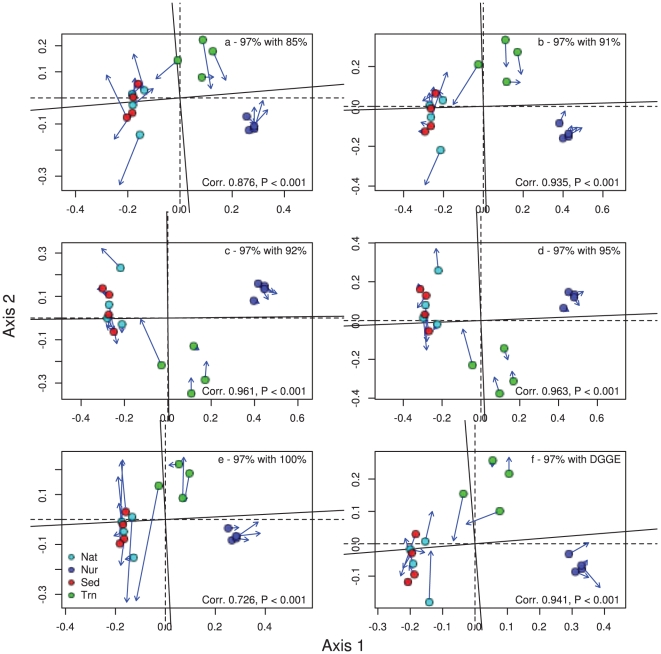
Ordination showing the first two PCO axes of the *Alphaproteobacteria* only analysis for a) 85% cut-off level with 97%, b) 91% cut-off level with 97%, c) 92% cut-off level with 97%, d) 95% cut-off level with 97%, e) 100% cut-off level and f) DGEE data with 97% cut-off level. The arrows in the ordination point to the target configuration, the actual symbols represent the rotated configuration, i.e., the ordination based on the 97% cut-off values. Correlation (Corr) and significance values are given in the lower right corner of each graph.

**Figure 3:**
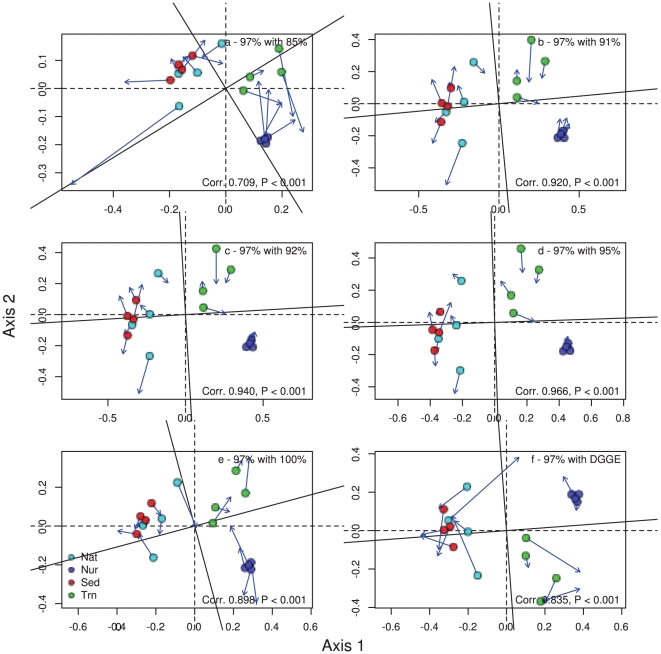
Ordination showing the first two PCO axes of the *Betaproteobacteria* only analysis for a) 85% cut-off level with 97%, b) 91% cut-off level with 97%, c) 92% cut-off level with 97%, d) 95% cut-off level with 97%, e) 100% cut-off level and f) DGEE data with 97% cut-off level. The arrows in the ordination point to the target configuration, the actual symbols represent the rotated configuration, i.e., the ordination based on the 97% cut-off values. Correlation (Corr) and significance values are given in the lower right corner of each graph.

The amount of variation in bacterial composition explained by microhabitats using DGGE profiles was 0.516 for *Bacteria*, 0.679 for *Alphaproteobacteria* and 0.586 for *Betaproteobacteria*. The amount of variation in composition explained by microhabitat based on pyrosequencing data followed a similar trend. The results varied from 0.250 at the 100% cut-off level to 0.638 at the 85% cut-off level for *Bacteria*, 0.243 at the 100% cut-off level to 0.582 at the 91% cut-off level for *Alphaproteobacteria* and 0.295 at the 100% cut-off level to 0.594 at the 92% cut-off level for *Betaproteobacteria*. Therefore, the compositional data based on DGGE fingerprinting and barcoded pyrosequencing at different cut-off levels (85–100%), revealed that both techniques showed highly significant differences in composition among microhabitats. All analyses revealed a primary gradient in composition along the first ordination axis from nursery samples to mangrove samples (Figs. 1, 2, 3). Samples of transplants (Trn) showed the greatest affinity with nursery samples using *Betaproteobacteria* and least affinity using all bacteria. There was a secondary gradient in composition along the second ordination axis from transplant samples to bulk sediment samples with native mangrove samples intermediate. This gradient was most pronounced using all bacteria at intermediate cut-off levels.

Ordination results obtained using different cut-off levels and DGGE were all similar to the 97% recommended cut-off level. Cut-off values from 91–95% in particular had highly significant (P<0.001; Figs. 1, 2, 3) correlations (>0.97 for all bacteria, >0.92 for *Alpha*- and *Betaproteobacteria*) with the 97% cut-off level. The 85% and 100% cut-off levels diverged, as expected, the greatest from the 97% level but still revealed the correlations were still highly significant (P<0.001 for all bacteria, *Alphaproteobacteria* and *Betaproteobacteria*). There were also highly significant correlations between the DGGE band data and 97% cut-off level data for all bacteria, *Alphaproteobacteria* and *Betaproteobacteria* (P<0.001).

## Discussion

In this study, we showed that bacterial composition differs among microhabitats using different methods (DGGE fingerprinting and barcoded pyrosequencing), taxa (all bacteria, *Alphaproteobacteria* and *Betaproteobacteria*) and, for pyrosequencing data, cut-off levels. These results agree with our previous study [17]. We also show that different cut-off levels (85–100%) and DGGE data yield significantly congruent results with the standard 97% cut-off level for pyrosequence data.

This congruence is important and demonstrates that DGGE fingerprinting data can be used as a good proxy for pyrosequence data when comparing bacterial composition. The advantages of DGGE compared to pyrosequencing are that it is orders of magnitude cheaper and much faster to analyse the samples and obtain results. DGGE fingerprinting, prior to pyrosequencing, provides a relatively cheap and rapid means of exploring compositional variation among samples and testing for variation in composition among previously defined groups of samples and is, as such, a complement to the more in depth pyrosequencing.

The congruence we have demonstrated confirms previous studies. In a comparison of bacterial and archaeal communities in fermented food, Roh et al. [9] showed that pyrosequencing and DGGE generally agreed in the (non)-detection of certain taxa. They did, however, note that DGGE failed to detect taxa found in the pyrosequencing analysis, which revealed more diverse bacterial communities. Using pyrosequencing, Pommier et al. [31] found that bacteria in surface samples collected near the coast were more diverse than open sea samples; coastal and open sea samples, furthermore, formed two distinct clusters in a non-metric multidimensional scaling ordination. Deep sea samples also clustered together. These results were previously observed in studies that used DGGE fingerprinting [32], [33]. Pommier et al. [31], interestingly, showed that ordination results using only 30 or 300 of the most abundant OTU's obtained with pyrosequencing gave essentially the same result as when all 3000 OTU's were used. This would explain the similarity of results obtained with DGGE, which tends to capture the most abundant species. However, it is important to note that, despite the highly congruent results, the DGGE analyses in this study targeted the hypervariable regions V6 and V9 of the 16S rRNA gene while the pyrosequence analyses targeted the V4 region of this gene [34]. The use of primers targeting the same hypervariable regions of the 16S rRNA gene (or any other phylogenetic marker) may provide even greater congruence.

Our results showed that the main gradient of variation in composition was between nursery and mangrove samples with samples taken from the rhizosphere of transplants intermediate in composition. Analyses (both DGGE fingerprinting and pyrosequencing) only using *Alphaproteobacteria* and, particularly, *Betaproteobacteria*, however, showed a much more pronounced affinity between nursery samples and transplants than analyses using all bacterial taxa. In addition to this, when using pyrosequencing data of all bacteria and to a lesser extent *Alphaproteobacteria*, there was a clear secondary gradient from transplants to bulk sediment samples, with native samples intermediate along the second ordination axis. This gradient was less apparent when using data obtained from *Betaproteobacteria* (pyroseqencing and DGGE fingerprint) and DGGE data of all taxa. Despite the congruence thus, evaluations based on different methods (DGGE or subsets of taxa) may lead to somewhat different conclusions about variation in composition.

In line with our previous study (17), the present study indicates that certain *Alphaproteobacteria* and *Betaproteobacteria* taxa appear to have been successful in remaining in the roots of the transplants after transplantation in the mangrove environment. The *Betaproteobacteria* analyses even show a greater affinity of the transplants to the nursery samples suggesting that most of these bacterial taxa were derived from the initial nursery conditions under which they were raised. This contrasts with most other bacterial higher level taxa given the much more pronounced affinity of transplant samples to other mangrove samples shown using all taxa (17).

In conclusion, we have demonstrated that both barcoded pyrosequencing and DGGE detected that different mangrove microhabitats harbour distinct communities of bacteria and show a marked congruence in results using different methods, taxa and cut-off levels. We suggest that DGGE fingerprinting data can be used as a proxy to ascertain the degree of compositional variation in bacterial communities and it provides a good quick and inexpensive method.

## Supporting Information

Figure S1 Denaturing gradient gel electrophoresis (DGGE) fingerprints of 16S ribosomal RNA gene fragments amplified from rhizospheres of nursery (Nur), transplanted (Trn), native *R. mangle* (Nat) and bulk sediment (Sed). All bacteria (a); *Alphaproteobacteria* (b); *Betaproteobacteria* (c). (M) Bacterial marker.
(TIF)

Figure S2 Principal coordinate (PCO) analysis. The first two axes of a PCO ordination are shown based on a matrix of OTU composition of all bacteria. Results are shown for various cut-off levels using pyrosequence data (a–f) and DGGE fingerprint data (g). The results of adonis analyses are shown in the lower right corner of each figure.
(TIF)

Figure S3 Principal coordinate (PCO) analysis. The first two axes of a PCO ordination are shown based on a matrix of OTU composition of *Alphaproteobacteria*. Results are shown for various cut-off levels using pyrosequence data (a–f) and DGGE fingerprint data (g). The results of adonis analyses are shown in the lower right corner of each figure.
(TIF)

Figure S4 Principal coordinate (PCO) analysis. The first two axes of a PCO ordination are shown based on a matrix of OTU composition of *Betaproteobacteria*. Results are shown for various cut-off levels using pyrosequence data (a–f) and DGGE fingerprint data (g). The results of adonis analyses are shown in the lower right corner of each figure.
(TIF)

Figure S5 Relative abundance of the most abundant bacterial classes () with the exception of the *Deferribacteres* class, which was slightly more abundant overall than the *Betaproteobacteria* class. Bars represent the mean relative abundance for each microhabitat and error bars represent a single standard deviation.
(TIF)
