# Supplementary figures and images for: Assessment of Variation in Bacterial Composition among Microhabitats in a Mangrove Environment Using DGGE Fingerprints and Barcoded Pyrosequencing
Source: PLoS One. 2012 Jan 11;7(1):e29380. doi: 10.1371/journal.pone.0029380 (PMC3256149; doi:10.1371/journal.pone.0029380)

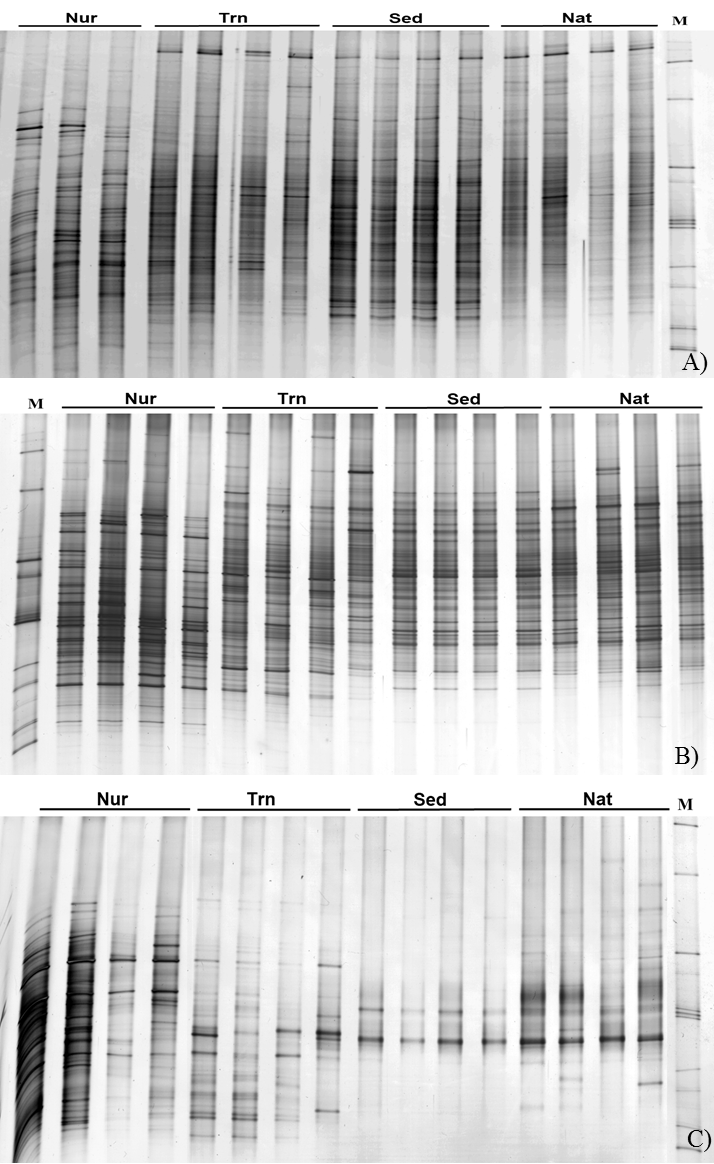

Supplement: Figure S1 — Denaturing gradient gel electrophoresis (DGGE) fingerprints of 16S ribosomal RNA gene fragments amplified from rhizospheres of nursery (Nur), transplanted (Trn), native R. mangle (Nat) and bulk sediment (Sed). All bacteria (a); Alphaproteobacteria (b); Betaproteobacteria (c). (M) Bacterial marker. (TIF) [file pone.0029380.s001.tif]

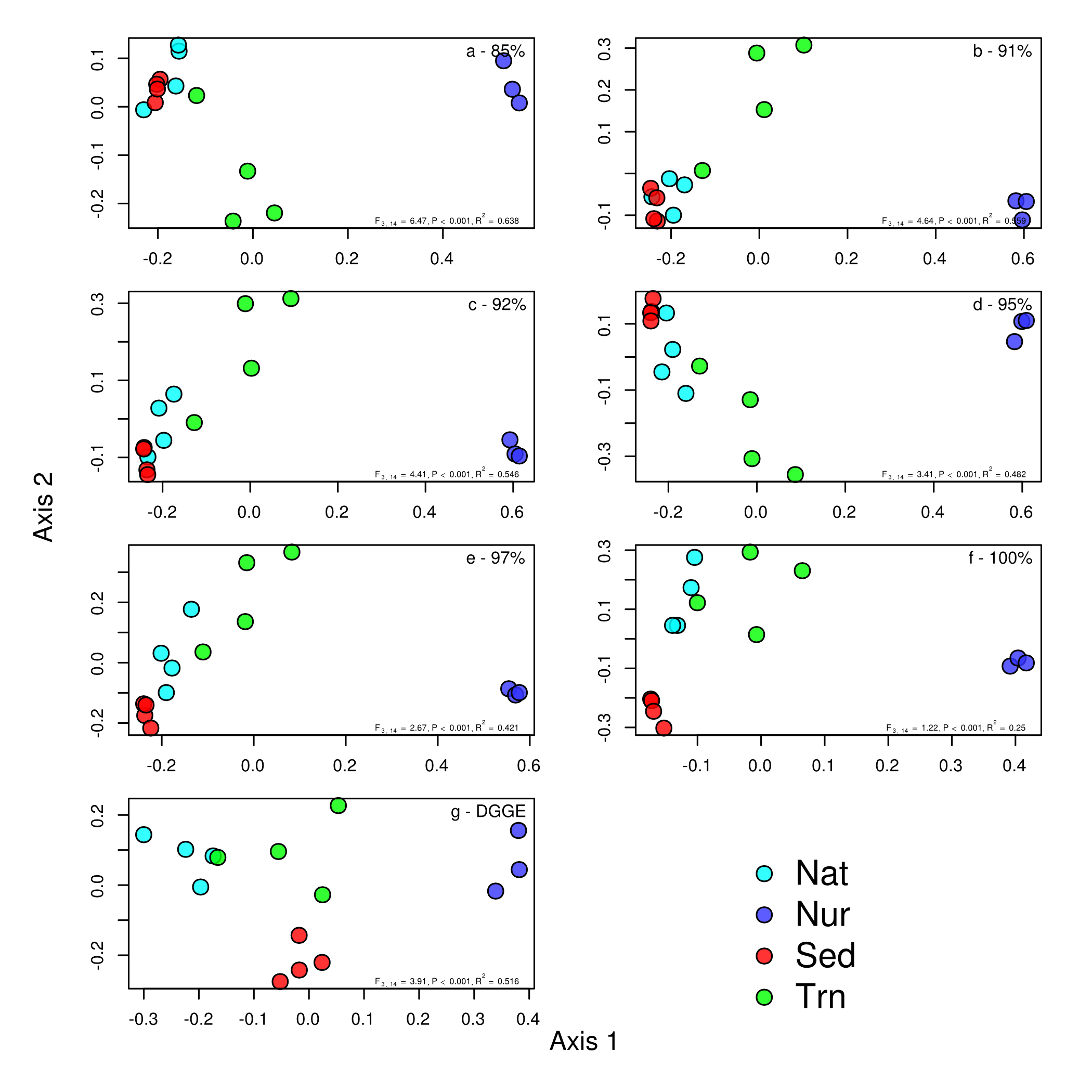

Supplement: Figure S2 — Principal coordinate (PCO) analysis. The first two axes of a PCO ordination are shown based on a matrix of OTU composition of all bacteria. Results are shown for various cut-off levels using pyrosequence data (a–f) and DGGE fingerprint data (g). The results of adonis analyses are shown in the lower right corner of each figure. (TIF) [file pone.0029380.s002.tif]

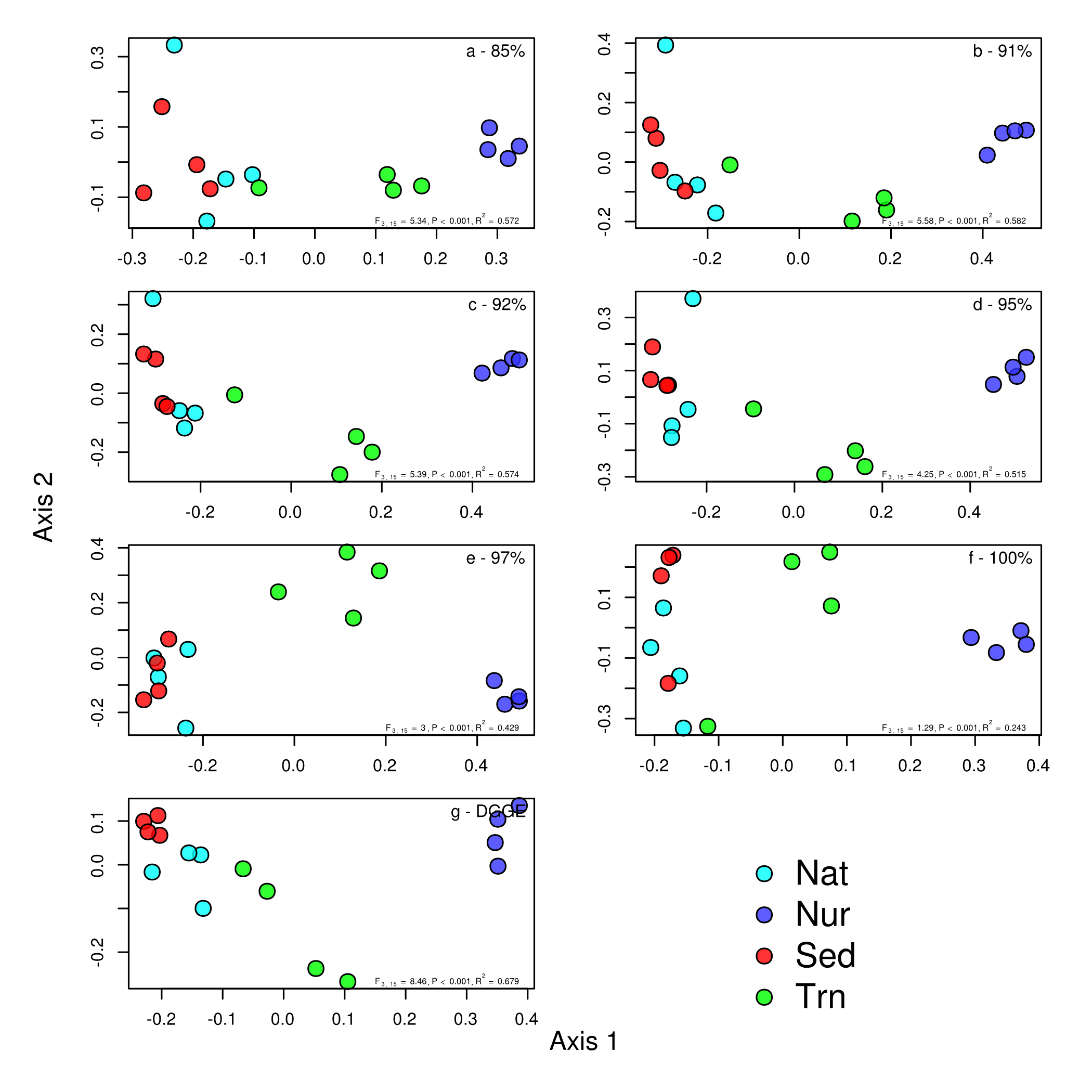

Supplement: Figure S3 — Principal coordinate (PCO) analysis. The first two axes of a PCO ordination are shown based on a matrix of OTU composition of Alphaproteobacteria. Results are shown for various cut-off levels using pyrosequence data (a–f) and DGGE fingerprint data (g). The results of adonis analyses are shown in the lower right corner of each figure. (TIF) [file pone.0029380.s003.tif]

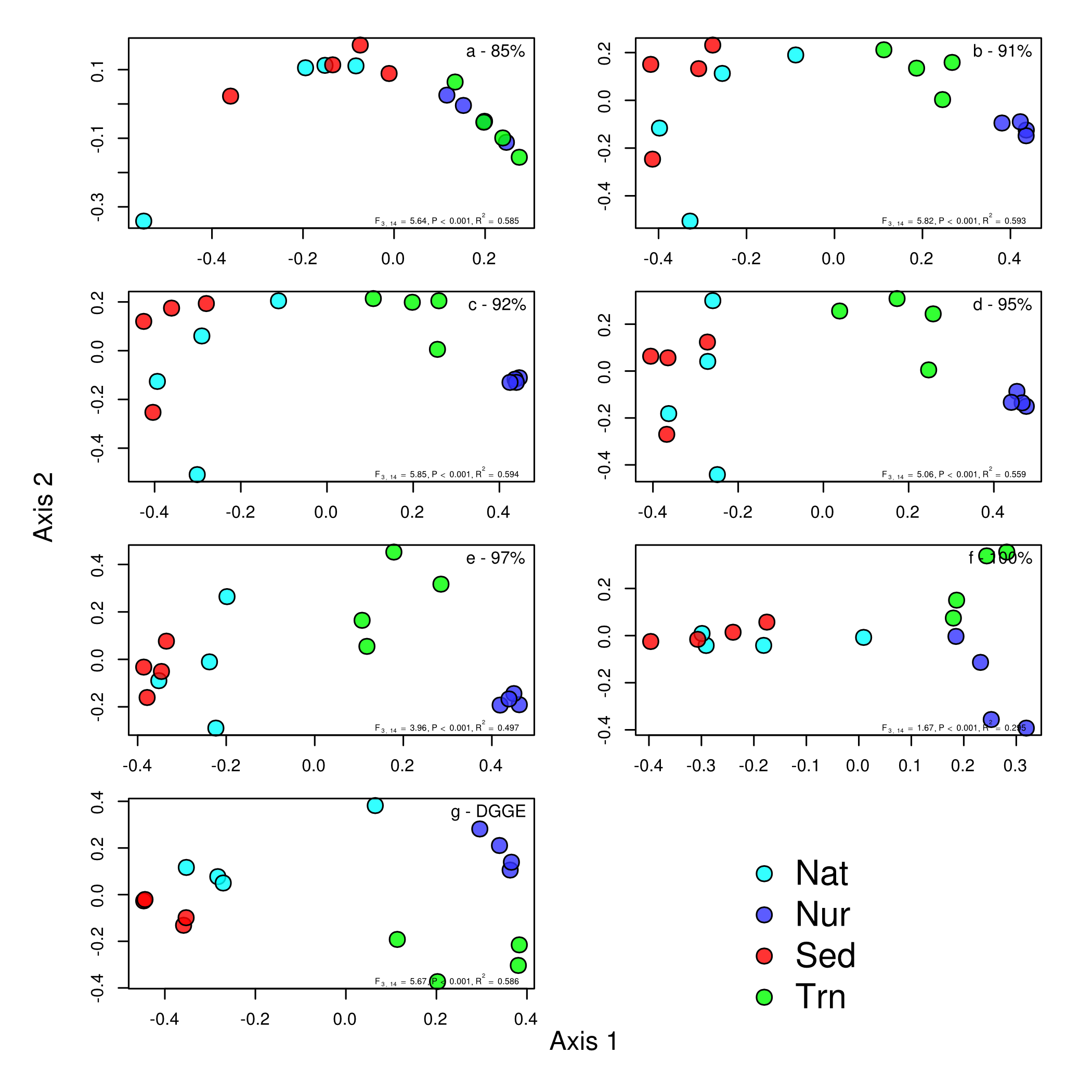

Supplement: Figure S4 — Principal coordinate (PCO) analysis. The first two axes of a PCO ordination are shown based on a matrix of OTU composition of Betaproteobacteria. Results are shown for various cut-off levels using pyrosequence data (a–f) and DGGE fingerprint data (g). The results of adonis analyses are shown in the lower right corner of each figure. (TIF) [file pone.0029380.s004.tif]

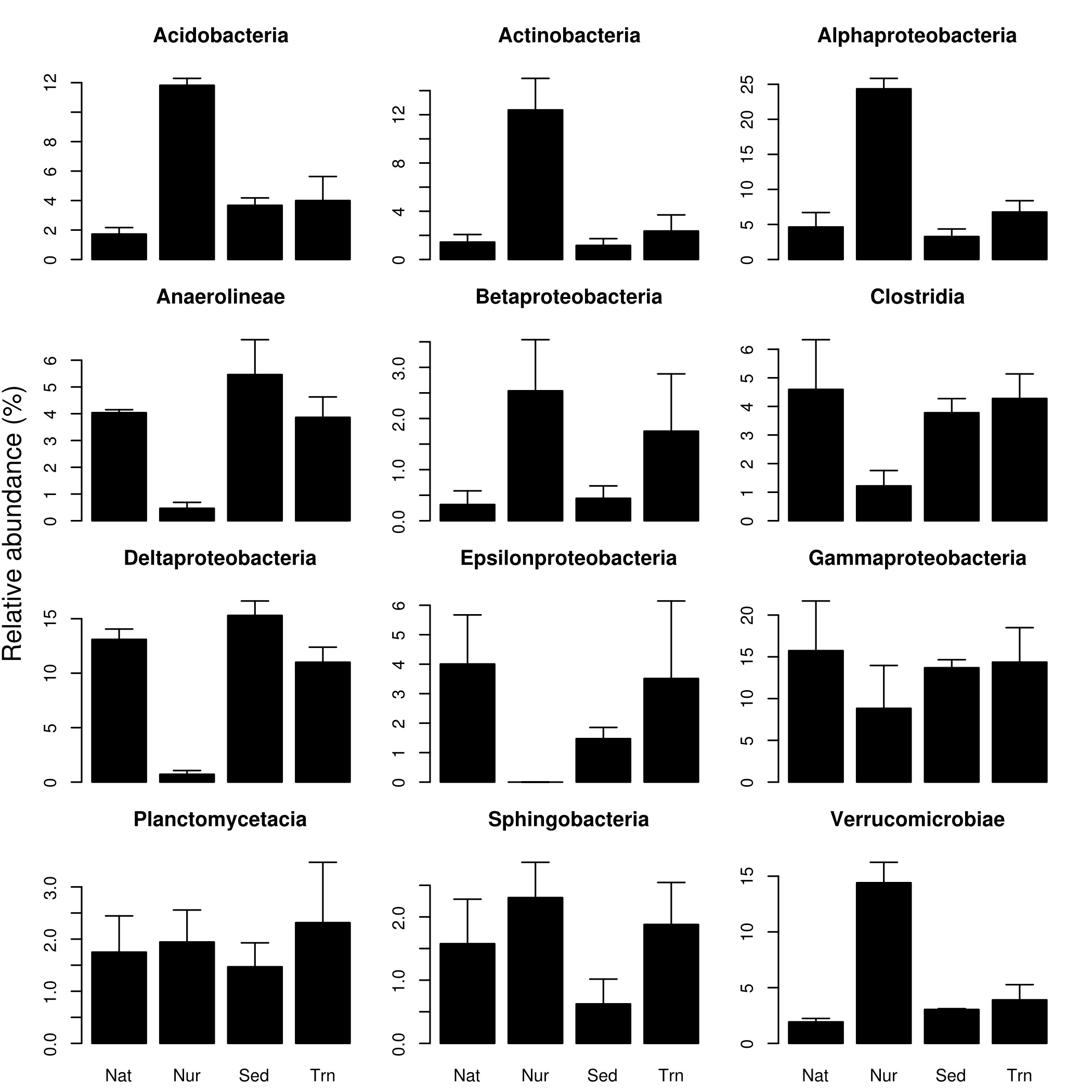

Supplement: Figure S5 — Relative abundance of the most abundant bacterial classes () with the exception of the Deferribacteres class, which was slightly more abundant overall than the Betaproteobacteria class. Bars represent the mean relative abundance for each microhabitat and error bars represent a single standard deviation. (TIF) [file pone.0029380.s005.tif]
